# COVID19-world: a shiny application to perform comprehensive country-specific data visualization for SARS-CoV-2 epidemic

**DOI:** 10.1186/s12874-020-01121-9

**Published:** 2020-09-21

**Authors:** Cristian Tebé, Joan Valls, Pau Satorra, Aurelio Tobías

**Affiliations:** 1 Biostatistics Unit, Institut d’Investigació Biomèdica de Bellvitge (IDIBELL), Hospitalet de Llobregat, 199 08908 L’Hospitalet de Llobregat, Barcelona, Spain; 2 Department of Mathematics, Universitat Autònoma de Barcelona (UAB), Bellaterra, Barcelona, Spain; 3 Institute of Environmental Assessment and Water Research (IDEA), Spanish Council for Scientific Research (CSIC), Barcelona, Spain

**Keywords:** SARS-CoV-2, COVID-19, Epidemic, Data visualization, Poisson regression, Mortality, Mortality rate, Case fatality rate, Basic reproduction number

## Abstract

**Background:**

Data analysis and visualization is an essential tool for exploring and communicating findings in medical research, especially in epidemiological surveillance.

**Results:**

Data on COVID-19 diagnosed cases and mortality, from January 1st, 2020, onwards is collected automatically from the European Centre for Disease Prevention and Control (ECDC). We have developed a Shiny application for data visualization and analysis of several indicators to follow the SARS-CoV-2 epidemic using ECDC data. A country-specific tool for basic epidemiological surveillance, in an interactive and user-friendly manner. The available analyses cover time trends and projections, attack rate, population fatality rate, case fatality rate, and basic reproduction number.

**Conclusions:**

The COVID19-World online web application systematically produces daily updated country-specific data visualization and analysis of the SARS-CoV-2 epidemic worldwide. The application may help for a better understanding of the SARS-CoV-2 epidemic worldwide.

## Background

The first confirmed case of SARS-CoV-2 in China was reported to the WHO country office in China on December 31st, 2019 [1]. The outbreak was declared a public health emergency of international concern on January 30th, 2020 [1]. Since then, up to June 17th, more than 210 countries have been affected worldwide, 8,162,276 people have been diagnosed, 443,685 have died due to the SARS-CoV-2 pandemic [2] and numbers are still growing.

Data analysis and visualization is an essential tool for exploring and communicating findings in medical research, especially in epidemiological surveillance [3]. It can help researchers and policymakers to identify trends that could be overlooked if the data were reviewed in tabular form. Here, we present the worldwide extension of a previous Shiny application for data visualization and analysis of several indicators to follow the SARS-CoV-2 epidemic in Spain [4]. With this extension, conceived as an independent tool, specific visualizations for any country worldwide can be produced to assess the time evolution of the pandemic, beyond the usual dashboards showing geographic variations [5]. Data is directly downloaded from the European Centre for Disease Prevention and Control (ECDC) each time that a user interacts with the application. Therefore, we have now developed the COVID19-World application, which systematically produces country-specific data visualization and analysis of trends and short-term projections for diagnosed cases and deaths (both for cumulative and incident data), case fatality rate, infection time, and basic reproduction number.

## Implementation

### Software

The COVID19-World application has been developed in *RStudio* [6], version 1.2.5033, using the *Shiny* package, version 1.4.0. Shiny offers the ability to develop a graphical user interface (GUI) that can be run locally or deployed online. Last is particularly beneficial to show and communicate updated findings to a broad audience. All the analyses have been carried out using R [7], version 3.6.3. The key R packages used in the tool implementation include *dplyr*, *xlsx,* and *vroom* for data management, *sjPlot,* and *EpiEstim* for data analysis, *shinydashboard*, *shinyFeedback*, *shinycssloaders,* and *kableExtra* for application enhancement and *plotly* for the graphical displays. The application is freely available online at [https://ubidi.shinyapps.io/covid19world], being the source code available under request through Github at [https://github.com/ubidi/covid19world]. Menus, tabs, and outputs are available in English, Spanish, and Catalan.

**Fig. 1:**
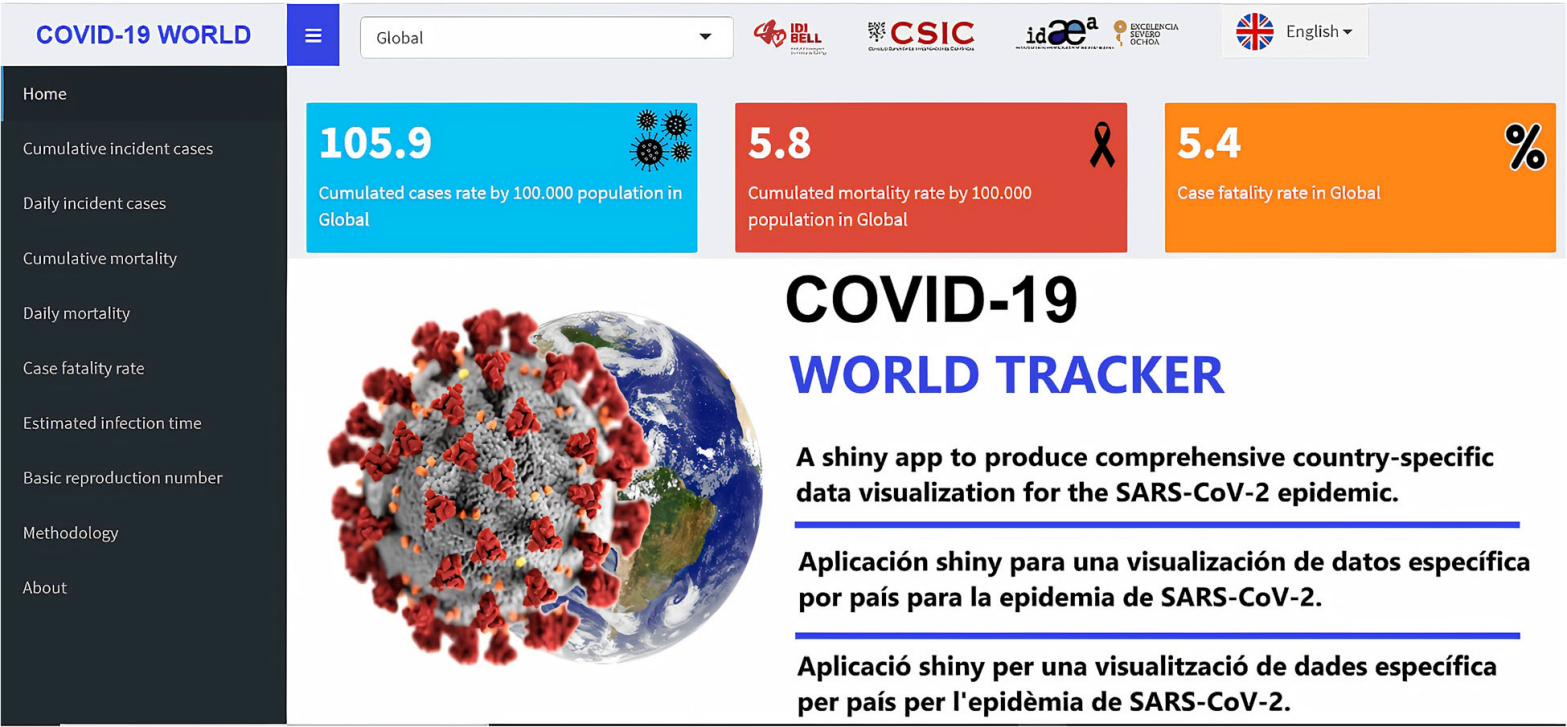
Home page of the COVID19-World application, for comprehensive country-specific data visualization for the SARS-CoV-2 epidemic. Available at [https://ubidi.shinyapps.io/covid19world/]

The European Centre for Disease Prevention and Control (ECDC) data file offers a downloadable file updated daily with the latest available public data on COVID-19 per day and country. Data is collected based on reports from health authorities worldwide by the ECDC’s Epidemic Intelligence team. The application has an automated process to update data and all analyses each time a user connects to the app. Data on COVID-19 diagnosed cases and mortality, from January 1st, 2020, onwards is collected at [https://www.ecdc.europa.eu/en/publications-data/download-todays-data-geographic-distribution-covid-19-cases-worldwide]. The downloadable dataset is updated daily and contains the latest available public data on COVID-19 worldwide. Countries with a population under 500,000 inhabitants are not included. The application is user friendly, based on intuitive menus to show data visualization for each of the analyses implemented, once a specific country has been chosen from the top dialog box (Fig. 1).

### Trends and projections

Trends for the number of diagnosed cases and deaths are estimated using Poisson regression models [8], allowing for over-dispersion [9]. A time-dependent polynomial function is used to estimate the expected number of cases. As the epidemic evolved the degree of the polynomial function was increased. The current model allows for a fourth-degree polynomial function, as follows:

$$ \log \left(\mathrm{E}\left({\mathrm{c}}_{\mathrm{t}}\right)\right)={\upbeta}_0+{\upbeta}_1\mathrm{t}+{\upbeta}_2{\mathrm{t}}^2+{\upbeta}_3{\mathrm{t}}^3+{\upbeta}_4{\mathrm{t}}^4 $$

where *t* = 1, 2, …, T, represents the time unit (from the first observed day until the last, T consecutive days in total), and *c*_*t*_ is the number of events. The estimated regression parameters and their standard errors are used to obtain the short-term projections, up to 3 days, and their 95% CI.

Nevertheless, these models are being regularly evaluated by checking the overdispersion parameter, the sum of Pearson residuals, and the deviance, in case a model reformulation with a better fit is necessary during the epidemic.

### Case fatality rate

The case fatality rate is defined as the ratio between the number of deaths and the number of diagnosed cases [10]. Thus, an offset is fitted into the Poisson regression model, also allowing for overdispersion, as the logarithm of the diagnosed cases:

$$ \log \left(\mathrm{E}\left({\mathrm{m}}_{\mathrm{t}}\right)\right)={\upbeta}_0+{\upbeta}_1\mathrm{t}+{\upbeta}_2{\mathrm{t}}^2+{\upbeta}_3{\mathrm{t}}^3+{\upbeta}_4{\mathrm{t}}^4+\log \left({\mathrm{c}}_{\mathrm{t}}\right) $$

where m_t_ is the daily number of deaths, and c_t_ is the daily number of diagnosed cases. Case fatality rates are also calculated for the same age groups.

We should acknowledge that it is not possible to make an accurate estimate of the case fatality rates due to the underreporting of cases diagnosed in official statistics [11]. Nonetheless, the estimation and monitoring of the case fatality rates monitoring are of especial interest in the current epidemic scenario.

### Infection time

Infection time, estimating the incubation period for COVID-19 between the interval of exposure to SARS-CoV-2 and the date of diagnosis is computed following the approach of Lauer et al. [12], who have recently analyzed the incubation period for COVID-19 in a cohort of symptomatic patients. For each patient, they collected the interval of exposure to SARS-CoV-2 and the date of appearance of symptoms. They assumed that the incubation time would follow, as in other viral respiratory tract infections, a Lognormal distribution.

$$ Lognormal\left( mu,{sigma}^2\right)= Lognormal\left(1.621,0.418\right) $$

We have replicated this distribution in the group of diagnosed cases to approximate the date of exposure to SARS-CoV-2 recursively:

$$ q(i)=\sum \limits_{j=1}^{14}P(j)\times {c}_{j+i} $$

where *p* is the number of diagnosed cases on a day *i*; *q* is the number of infected cases on day *i-j*; *j* = 1, 2, …, 14 is the maximum time it is expected that the disease can develop; and *P(j)* is the probability of presenting symptoms on day *j* according to a Lognormal probability distribution with the parameters defined by Lauer et al. [12]

To estimate the last 14 days, since the information on the diagnosed cases was not available for the forthcoming days, a fourth-degree polynomial model was used to project diagnosed cases. These latest estimates are displayed in the application with a different color.

### Basic reproduction number

The basic reproduction number (R_0_) is the average number of secondary cases of disease caused by a single infected individual over his or her infectious period [13]. This statistic, which is time and situation-specific, is commonly used to characterize pathogen transmissibility during an epidemic. The monitoring of R_0_ over time provides feedback on the effectiveness of interventions and on the need to intensify control efforts. The goal of control efforts is to reduce the R_0_ below the threshold value of 1 and as close to 0 as possible to control the epidemic. Here, we used the R package *EpiEstim* to estimate the basic reproduction number through the Wallinga and Teunis method [13], which assumes a gamma distribution for the serial interval. The serial interval is the time between the onset of symptoms in a primary case and the onset of symptoms of secondary cases, which is needed to estimate R_0_ throughout the epidemic. The mean and standard deviation of the serial interval distribution can vary depending on the disease [13]. Recently, Nishiura et al. [14] estimated a mean and standard deviation for the COVID-19 serial interval distribution of 4.7 and 2.9 days, respectively, being these the values we are using in our analysis for the gamma a priori distribution.

The goodness of fit of estimated models is evaluated to provide a better fit of the data during the epidemic. A deviance analysis is performed to compare the model’s fit. Moreover, to quantify the model error Poisson overdispersion parameter, the sum of Pearson’s residuals and deviances are shown.

## Results

**Fig. 2:**
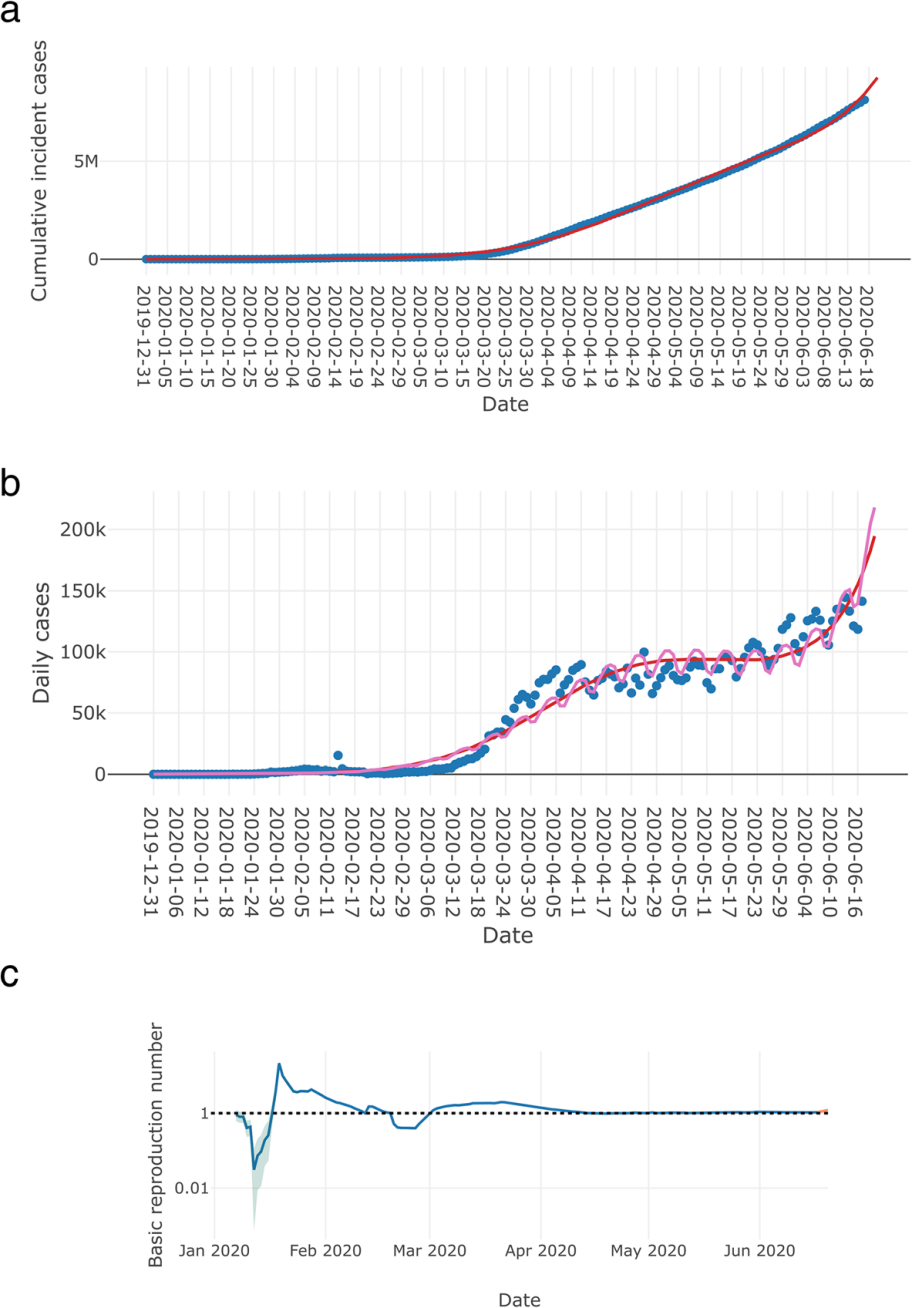
Standard output display of the COVID19-World application (results updated to June 17th, 2020) trend analysis and its 3-day projection at the global scale of cumulate incident cases (**a**); daily incident cases (**b**); and basic reproduction number (**c**)

**Fig. 3:**
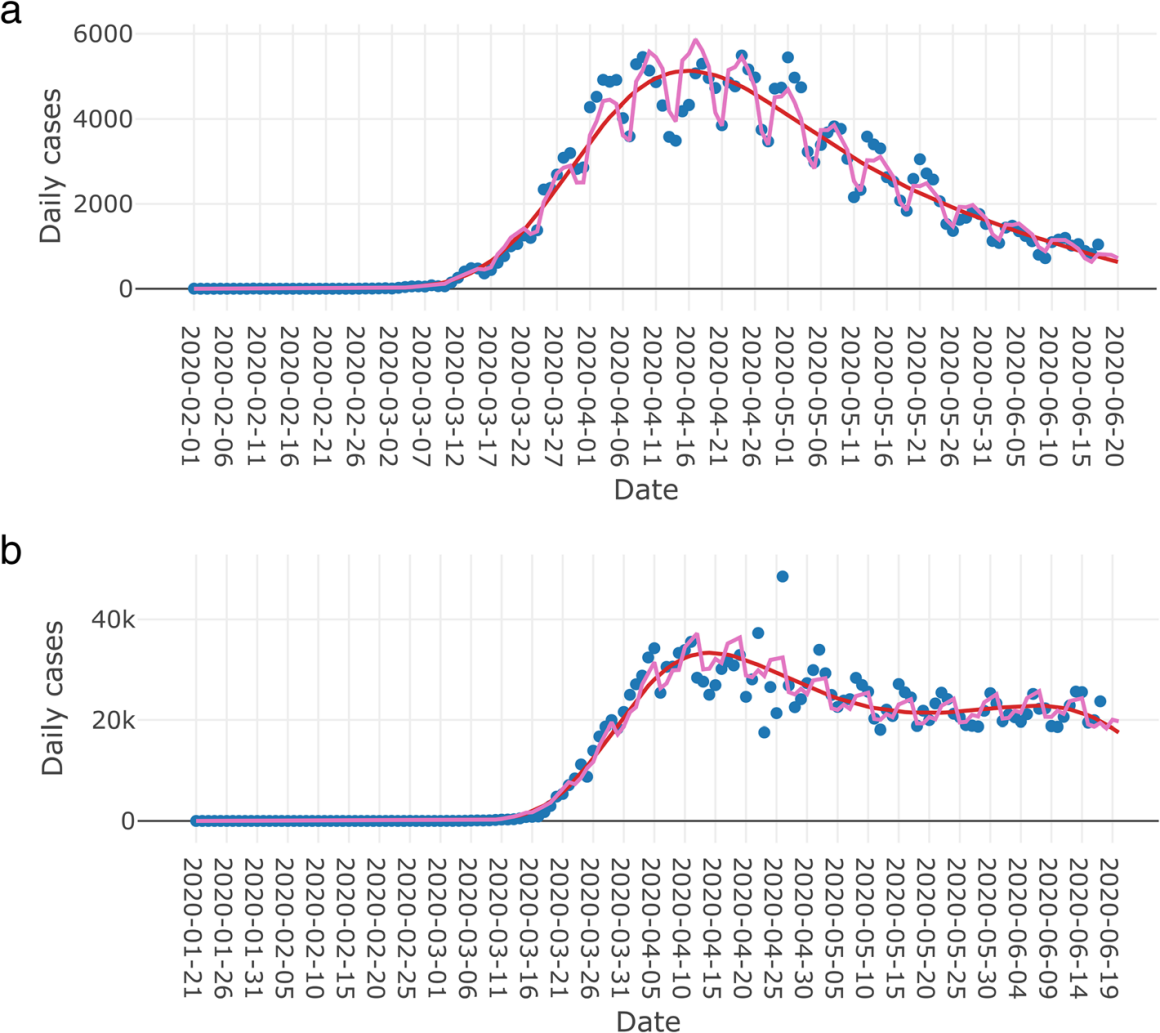
Propagated epidemic patron in the United Kingdom (**a**) and the United States (b)

Up to June 17th, an exponential increase in the global number of COVID-19 cases was observed (Fig. 2a and b). The shape of the curve on daily incident cases and mortality curves in most countries shows a propagated epidemic pattern (for example the United Kingdom and the United States in Fig. 3a and b). In a propagated outbreak, there is no common source and the outbreak spreads from person to person. The classic propagated curve has a series of progressively taller peaks, each an incubation period apart, which may lead to multiple waves of infection if secondary cases occur. However, in the case of SARS-Cov-2, waves of cases seem to overlap due to high viral transmission and the curve is more bell-shaped with a large cue. In most countries, the successive waves involve more and more people, until lockdown or social distancing measures were implemented.

**Fig. 4:**
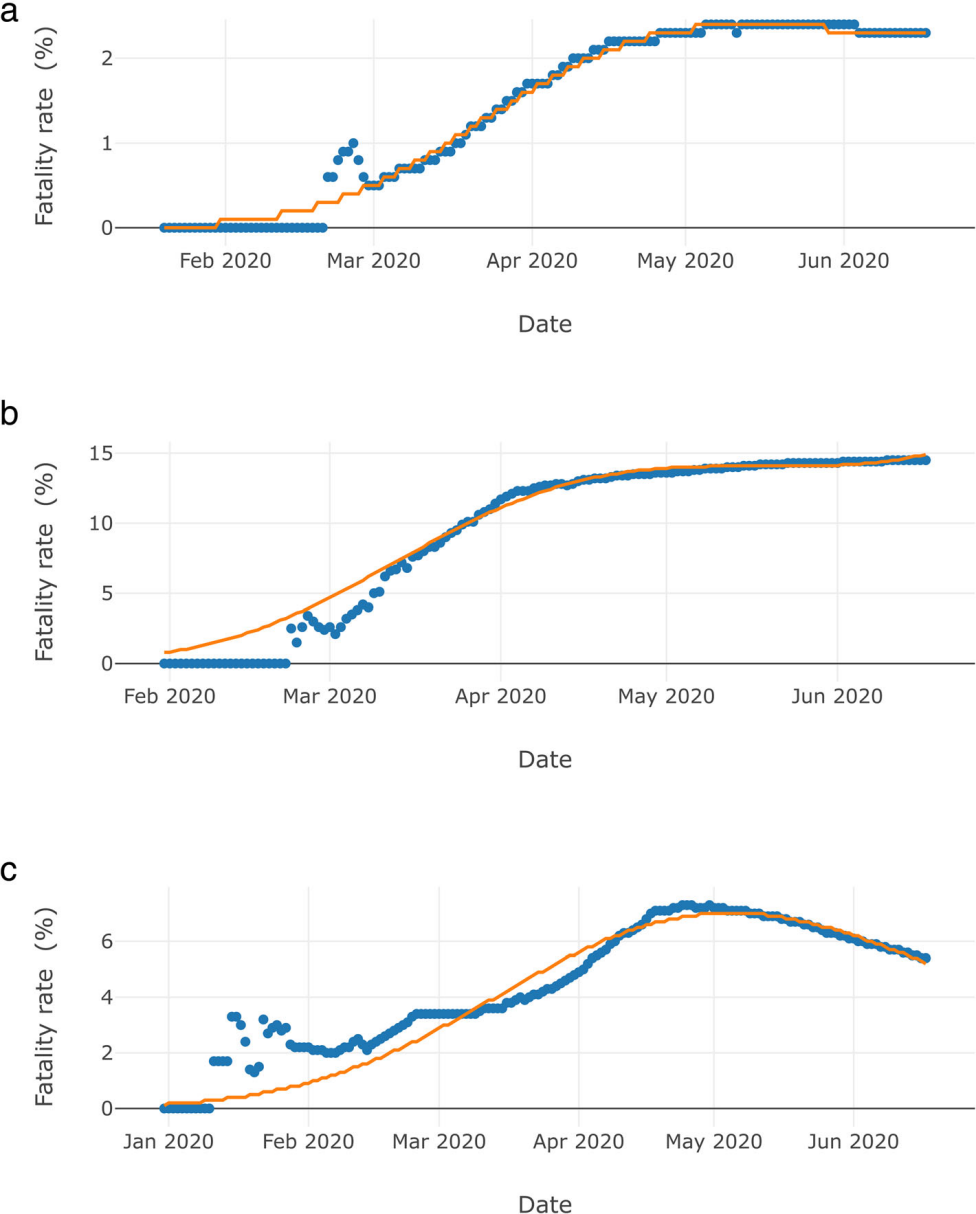
A variation on the case fatality rate South Korea (**a**), Italy (**b**), and globally (**c**)

In the counterpart, the case fatality rate ranges from 2% of South Korea to 15% of Italy and globally is 5.5% (Fig. 4a, b, and c respectively). This variation on the case fatality rate, in our opinion, could be explained by variability in the testing effort of each country to identify cases.

The estimated infection time has been globally reduced over the epidemic period (Fig. 2c), and the global basic reproductive number is currently around 1, except in those countries who are experimenting with a second wave.

## Discussion

The application allows the use of epidemiological indicators to a better understanding of the evolution of the COVID-19 epidemic in 167 countries affected. However, we should note that it has been developed mainly for descriptive purposes. Therefore, we avoided using complex mathematical models to predict an uncertain future with uncertain data [15]. We choose to use a classical approach fitting Poisson regression models, commonly used in epidemiological research [8, 16], which not only is parsimonious but also showed reasonable goodness of fit. To go beyond epidemiological information, we add some tabs with relevant statistical information about estimated models. The target audience of this web application is wide. It includes scientists, researchers, policymakers, journalists, and the general public with a special concern on the SARS-CoV-2 epidemic. Since publication to date, our application had received more than 6 thousand visits from all around the world, but mainly from Europe and the United States.

Tracking the SARS-CoV-2 epidemic spread has become a topic of great interest. There are several apps, dashboards, and maps on the internet showing local, national, and international figures. Among the most popular the Johns Hopkins University web offers a worldwide view on a map with the cumulate number of confirmed cases, deaths, and recovered worldwide and by country and state/region, but not in all countries. The World Health Organization has its dashboard showing more or less the same information on confirmed cases and deaths by country. Other applications focus its figures on predictions. Is the case of the web developed by the Institute for Health Metrics and Evaluation (IHME) of the University of Washington. On its web, you can see current data and projections on cumulate and daily cases and deaths in the United States and the European Economic Area. Moreover, they used a mixed-effects non-linear regression framework to estimate the trajectory of the cumulative and daily death rate as a function of the implementation of social distancing measures.

The choice of reliable data sources is complex and not absent from controversy [17, 18]. Our main data source is provided daily by the ECDC (19) and we mainly chose epidemiological indicators based on those which could be derived from diagnosed cases and daily deaths.

We should acknowledge that the application does not take into account the changes in the definition of a case diagnosed by COVID-19, nor the population exposed. So, the number of events is modeled directly instead of the incidence rate, assuming that the entire population is at risk, except for the case fatality rate. Moreover, the analyses are not free from the biases linked to the source of information provided by the ECDC. For example, in several countries, it can take some weeks until death certificates are available in databases. Hence, estimates might not be accurate due to the underreporting of diagnosed cases and mortality in official statistics.

## Conclusions

The COVID19-World is a web-based application that comes to fill a gap, presenting a set of useful tools for updated country-specific analysis and visualization of epidemiological indicators of the COVID-19 epidemic worldwide. The web application facilitates a better understanding of the evolution of the epidemic in each country and might be useful for country-specific epidemiological surveillance.

### Availability and requirements

Project name: COVID-19 WORLD.

Project home page: https://ubidi.shinyapps.io/covid19world/

Operating system: Platform independent.

Programming language: R.

Other requirements: Any web browser, or RStudio with libraries shiny, dplyr, xlsx, vroom, sjPlot, EpiEstim, shinydashboard, shinyFeedback, shinycssloaders, and kableExtra.

License: published under the GNU General Public License Version 2.

Any restrictions to use by non-academics: Commercial organizations are welcome to contact the author prior to use.

## Data Availability

Data is available at ECDC “Download today’s data on the geographic distribution of COVID-19 cases worldwide” https://www.ecdc.europa.eu/en/publications-data/download-todays-data-geographic-distribution-covid-19-cases-worldwide. Source code is available under request through Github at [https://github.com/ubidi/covid19world].

## Abbreviations

COVID-19: Coronavirus disease 2019
GLP: General public license
GNU: GNU’s not unix!
GUI: Graphical user interface
SARS-CoV-2: Severe acute respiratory syndrome coronavirus 2

## References

[1] Organization WH. Rolling updates on coronavirus disease (COVID-19). Updated 13 May 2020 Geneva (updated May 13th, 2020). [https://www.who.int/emergencies/diseases/novel-coronavirus-2019/events-as-they-happen]. Accessed May 2020.

[2] Roser M, Ritchie H, Ortiz-Ospina E. Hasell J. Coronavirus Pandemic (COVID-19); 2020. Published online at OurWorldInData.org. Retrieved from: https://ourworldindata.org/coronavirus. [Online Resource].

[3] Carroll LN, Au AP, Detwiler LT, Fu TC, Painter IS, Abernethy NF (2014). Visualization and analytics tools for infectious disease epidemiology: a systematic review. J Biomed Inform.

[4] Valls J, Tobias A, Satorra P, Tebe C. COVID19-tracker: a shiny app to analise data on SARS-CoV-2 epidemic in Spain. Gac Sanit. 2020;S0213-9111(20):30085–6. 10.1016/j.gaceta.2020.04.002. 10.1016/j.gaceta.2020.04.002 PMC7184003 32417117

[5] Dong E, Du H, Gardner L (2020). An interactive web-based dashboard to track COVID-19 in real time. Lancet Infect Dis.

[6] Team R (2015). RStudio: integrated development for R.

[7] Team RC (2020). R: a language and environment for statistical computing.

[8] Dyba T, Hakulinen T (2000). Comparison of different approaches to incidence prediction based on simple interpolation techniques. Stat Med.

[9] Payne EH, Hardin JW, Egede LE, Ramakrishnan V, Selassie A, Gebregziabher M (2017). Approaches for dealing with various sources of overdispersion in modeling count data: scale adjustment versus modeling. Stat Methods Med Res.

[10] Rothman K, Greenland S (1998). Modern epidemiology.

[11] Battegay M, Kuehl R, Tschudin-Sutter S, Hirsch HH, Widmer AF, Neher RA (2020). 2019-novel coronavirus (2019-nCoV): estimating the case fatality rate - a word of caution. Swiss Med Wkly.

[12] Lauer SA, Grantz KH, Bi Q, Jones FK, Zheng Q, Meredith HR (2020). The incubation period of coronavirus disease 2019 (COVID-19) from publicly reported confirmed cases: estimation and application. Ann Intern Med.

[13] Cori A, Ferguson NM, Fraser C, Cauchemez S (2013). A new framework and software to estimate time-varying reproduction numbers during epidemics. Am J Epidemiol.

[14] Nishiura H, Linton NM, Akhmetzhanov AR (2020). Serial interval of novel coronavirus (COVID-19) infections. Int J Infect Dis.

[15] Barton CM, Alberti M, Ames D, Atkinson JA, Bales J, Burke E (2020). Call for transparency of COVID-19 models. Science.

[16] Kuhn L, Davidson LL, Durkin MS (1994). Use of Poisson regression and time series analysis for detecting changes over time in rates of child injury following a prevention program. Am J Epidemiol.

[17] Pearce N, Vandenbroucke JP, VanderWeele TJ, Greenland S. Accurate statistics on COVID-19 are essential for policy guidance and decisions. Am J Public Health. 2020;110(7):949–51. 10.2105/AJPH.2020.305708. Epub 2020 Apr 23. 10.2105/AJPH.2020.305708 PMC7287551 32324422

[18] Wolkewitz M, Puljak L (2020). Methodological challenges of analysing COVID-19 data during the pandemic. BMC Med Res Methodol.

